# Supplementary material for: Monkeypox virus genomic accordion strategies
Source: Nat Commun. 2024 Apr 18;15:3059. doi: 10.1038/s41467-024-46949-7 (PMC11026394; doi:10.1038/s41467-024-46949-7)
Supplement: Supplementary file 1 — Supplementary Information [file 41467_2024_46949_MOESM1_ESM.pdf]

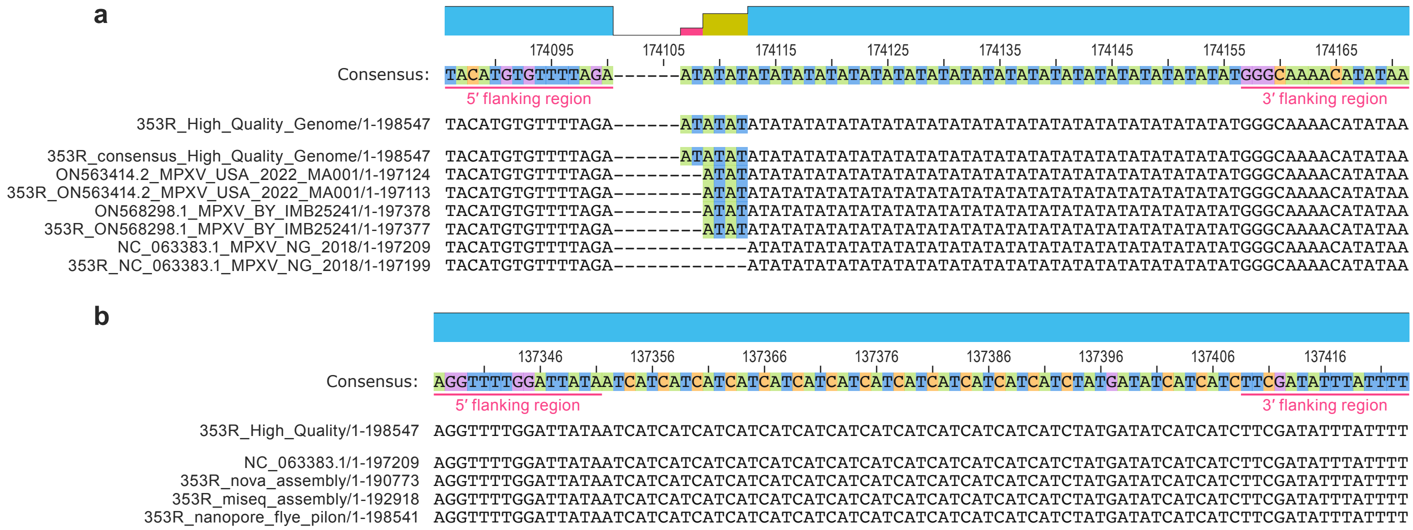

Figure S1. Examples for read mapping artefacts and correction in monkeypox virus (MPXV) genome low-complexity regions (LCRs)
a LCR2 alignment highlighting differences compared with various consensus sequences. b LCR7 alignment demonstrating identical results obtained using three sequencing platforms compared to the subclade IIb lineage A MPXV reference isolate MPXV-M5312_HM12_Rivers sequence.

a

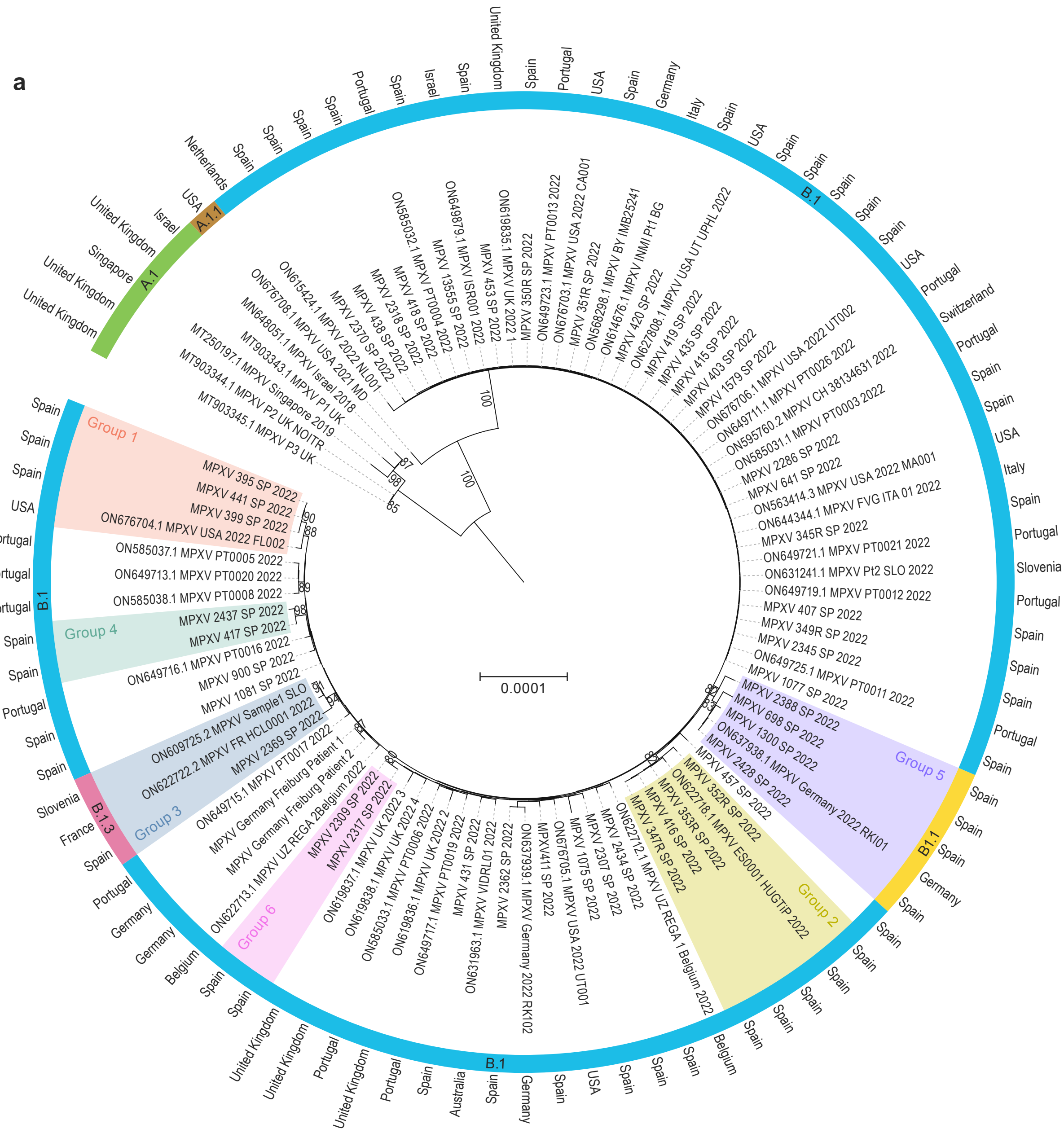

Figure S2. Phylogenetic analysis of monkeypox virus (MPXV)

a Phylogenetic maximum-likelihood (ML) tree showing monkeypox virus (MPXV) subclade IIb single-nucleotide polymorphism (SNP) clustering. Bootstrap supports >60 are indicated by labels with their number of supports. b Haplotype network showing SNP differences among samples included in the phylogenetic tree. Details on groups can be found in Supplementary Data 4.

b

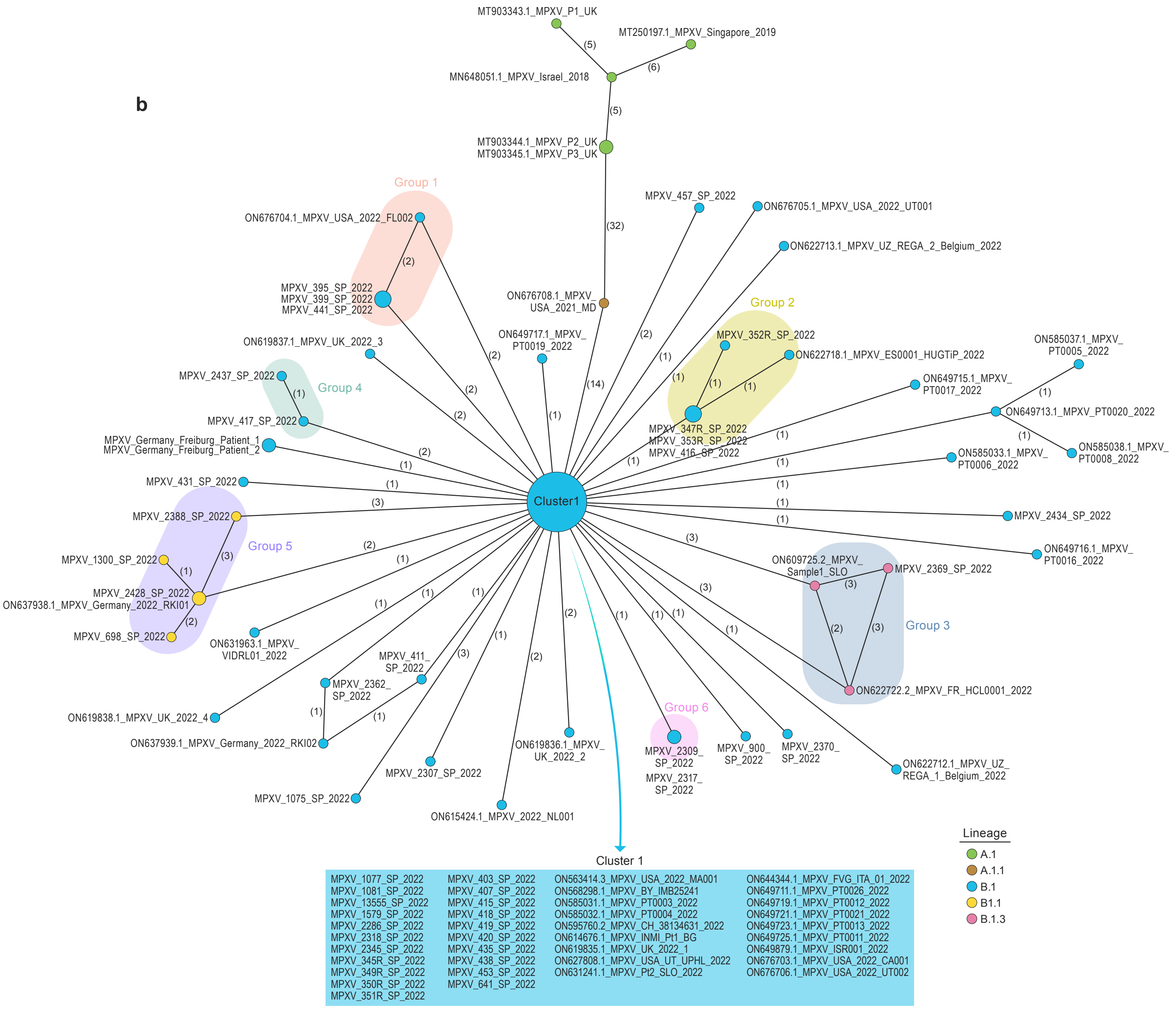

Figure S2. Phylogenetic analysis of monkeypox virus (MPXV)
a Phylogenetic maximum-likelihood (ML) tree showing monkeypox virus (MPXV) subclade IIb single-nucleotide polymorphism (SNP) clustering. Bootstrap supports >60 are indicated by labels with their number of supports. b Haplotype network showing SNP differences among samples included in the phylogenetic tree. Details on groups can be found in Supplementary Data 4.

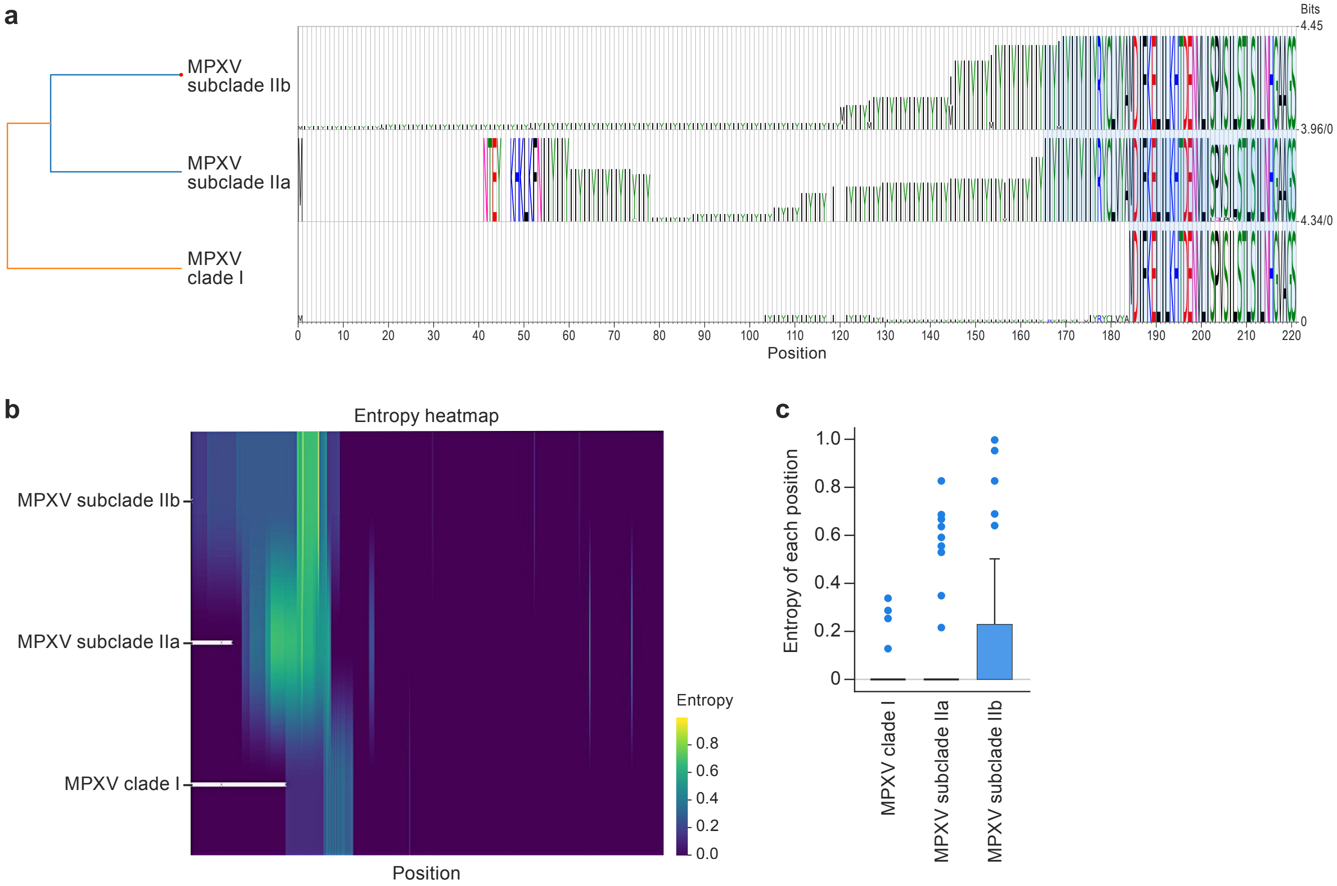

Figure S3. Conservation and variation in proteins encoded by orthologous poxvirus gene (OPG) 208
a MetaLogo visualization of conserved and varying amino-acid residues in OPG-encoded proteins among monkeypox virus (MPXV) clade I, subclade IIa, and subclade IIb, with homologous and nonhomologous sites highlighted; b Entropy heatmap; c Entropy analysis by site

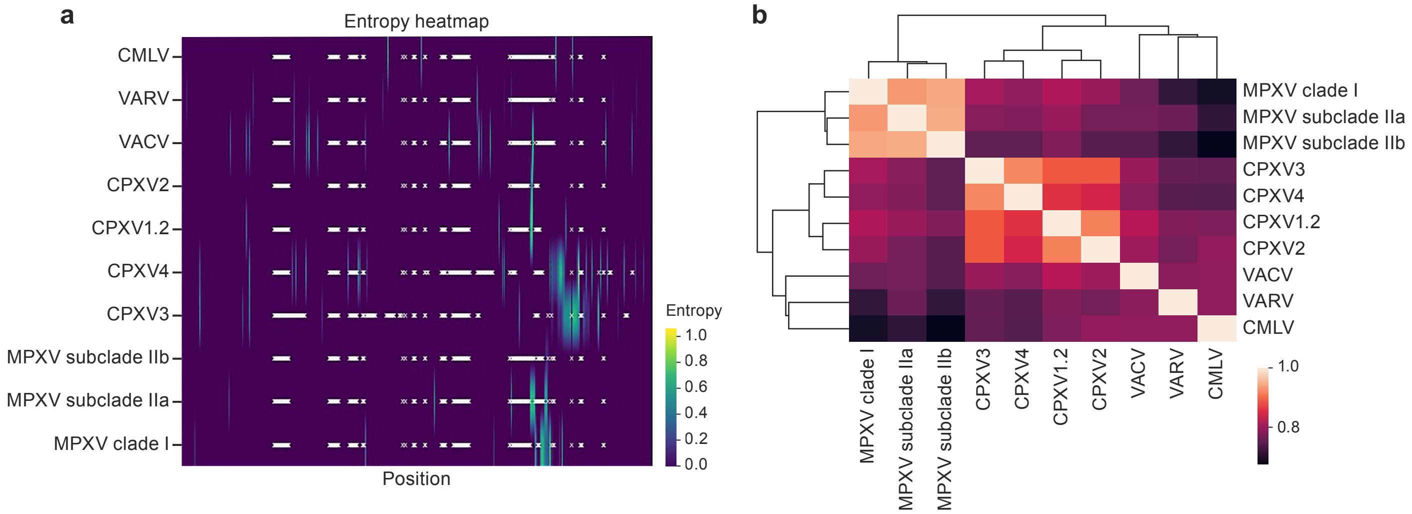

Figure S4. Conservation and variation in proteins encoded by orthologous poxvirus gene (OPG) 153
a Entropy heatmap. CMLV, camelpox virus; VARV, variola virus; VACV, vaccinia virus; CPXV, cowpox virus; MPXV, monkeypox virus. b Clustering result of sequence logo groups. MPXV, monkeypox virus; CPXV, cowpox virus; VACV, vaccinia virus; VARV, variola virus; CMLV, camelpox virus.

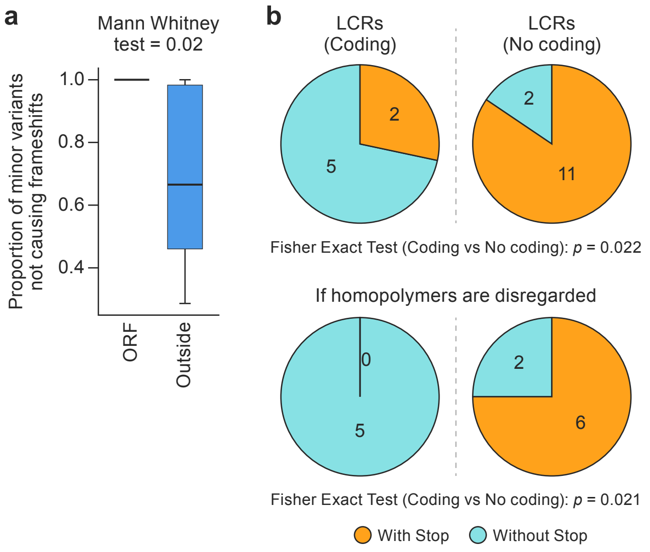

Figure S5. Low-complexity regions (LCRs) in coding areas evolve mostly as changes of nucleotide triplets (codons), a pattern significantly different from LCRs in non-coding areas

a Proportion of variants in LCRs (inside versus outside coding areas) that do not cause frameshifts. b Proportion of LCRs (inside versus outside coding areas) that include stop codons.

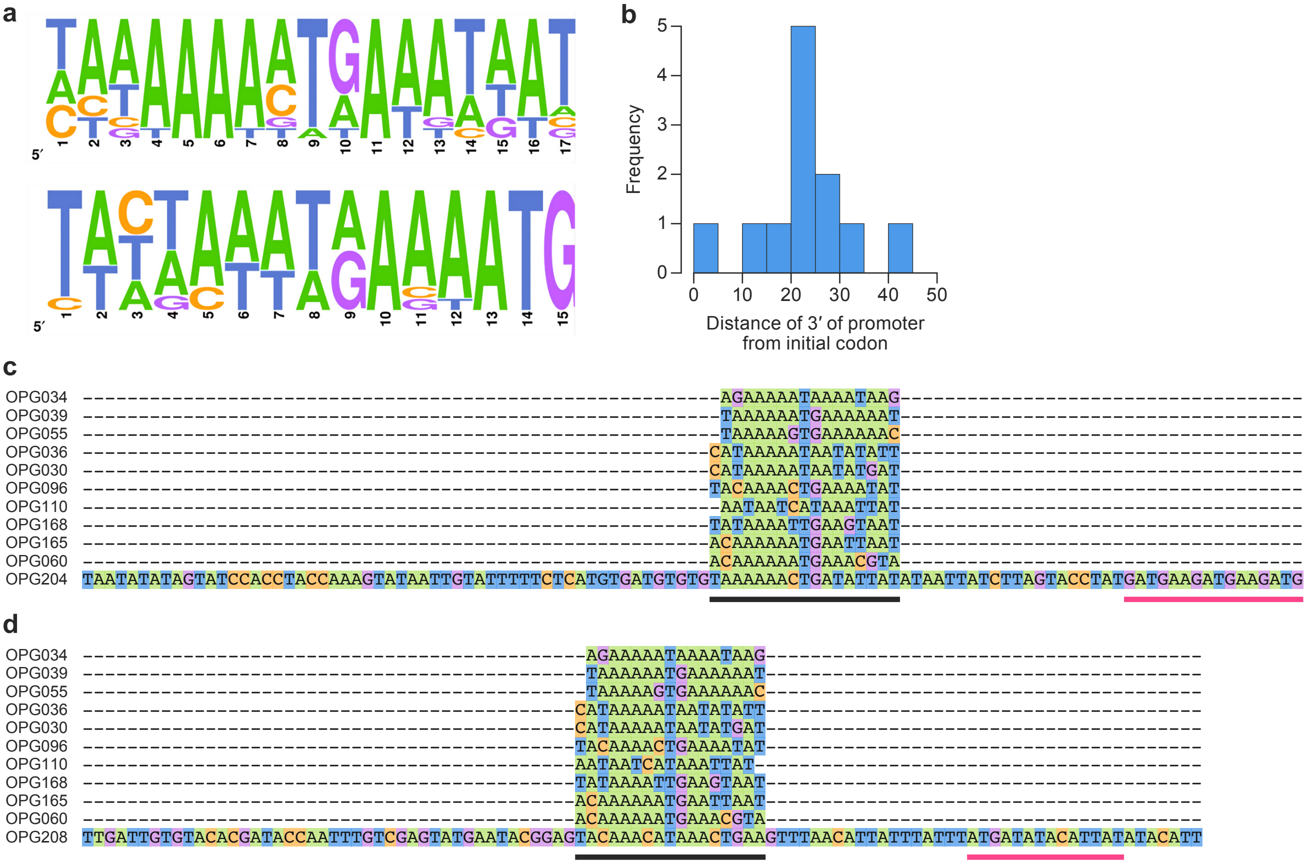

Figure S6. Characterization of monkeypox virus (MPXV) promoters

a Logo plot displaying the frequencies of nucleotides that form the promoter sequences from the top 10 genes most expressed early (above) and late during infection (below). b Distribution (top 10 early genes) of distances of the 3' of the promoter to the initial codon (note that the promoter of late genes includes the first alternative start codon [ATG]). c 5' end of OPG204, including its promoter aligned to the top 10 early genes (black box) and the LCR21 repeat region, that contains its ATG (red box). d 5' end of OPG208, including its promoter aligned to the top 10 late genes (black box) and the ATG followed by the first two repeats of LCR3 (red box).

SUPPLEMENTARY NOTE 1

Supplementary Note 1. Analysis parameters for validation of short tandem repeats (STRs) in National Center for Biotechnology Information (NCBI) Sequence Read Archive (SRA)

A. Short tandem repeats curation

Short Tandem Repeats areas were manually inspected in assemblies from sample 353R. Alignments show for all identified LCRs:

1. NC_063363.1 reference genome
2. 353_R sample de novo assembly from MiSeq reads 2x 300
3. 353_R sample de novo assembly from NovaSeq 2x150 reads
4. 353_R sample de novo assembly from Nanopore reads.
5. High quality genome

LCR1:

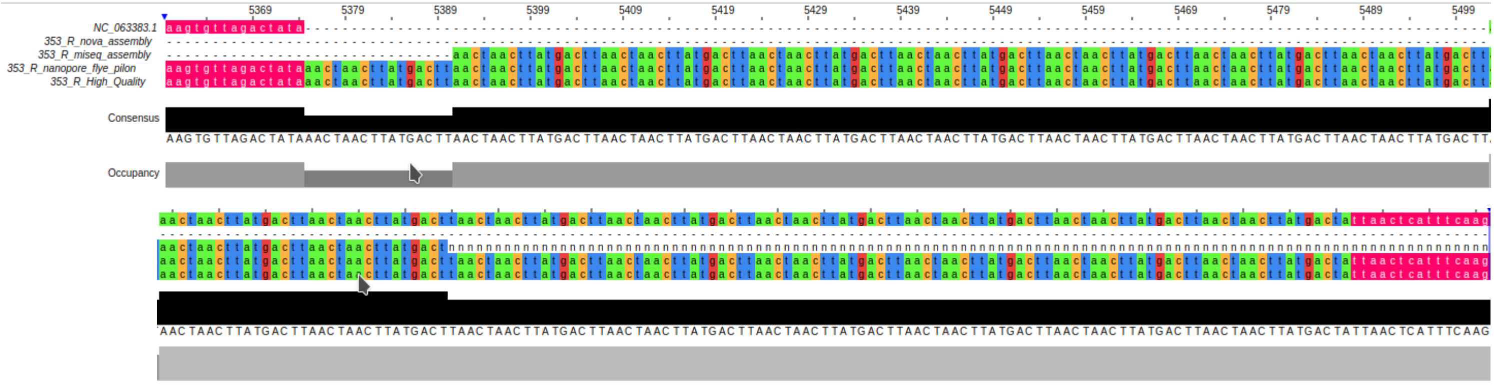

LCR2:

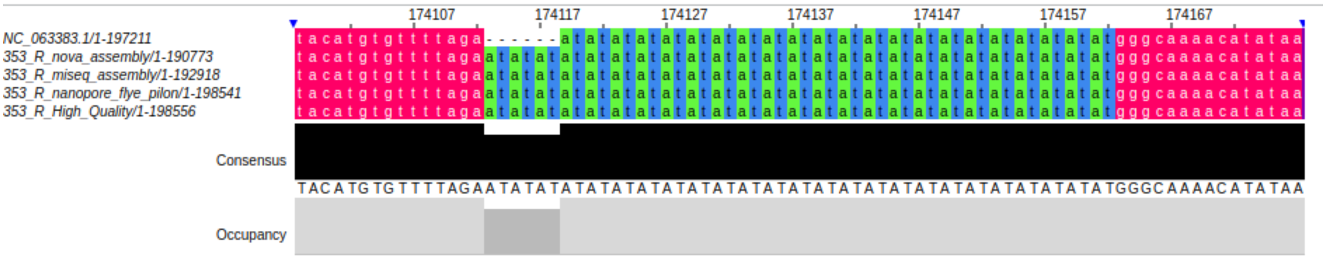

LCR3:

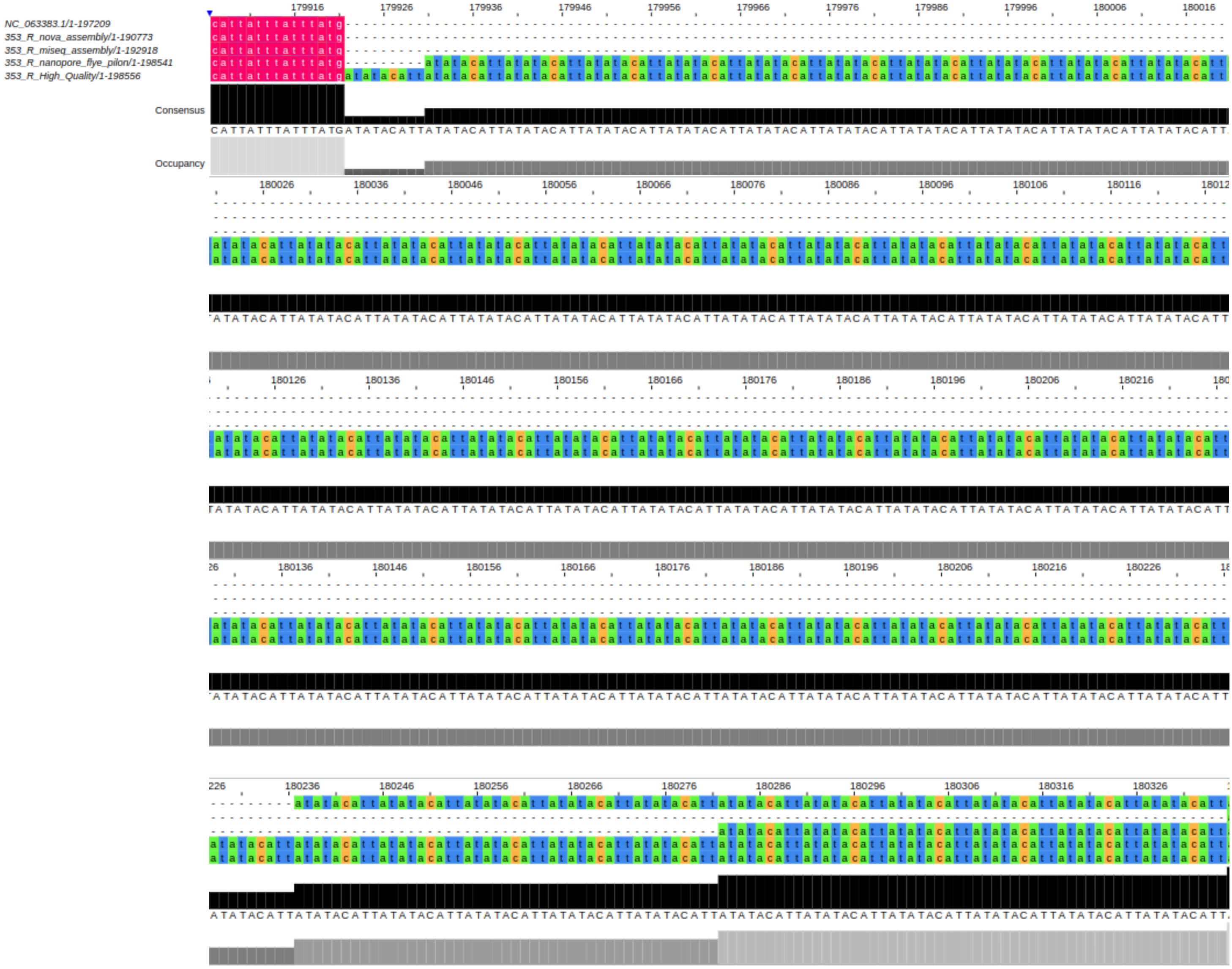
NC_063383.1/1-197209
353_R_nova_assembly/1-190773
353_R_miseq_assembly/1-192918
353_R_nanopore_flye_pilon/1-198541
353_R_High_Quality/1-198556
Consensus
Occupancy

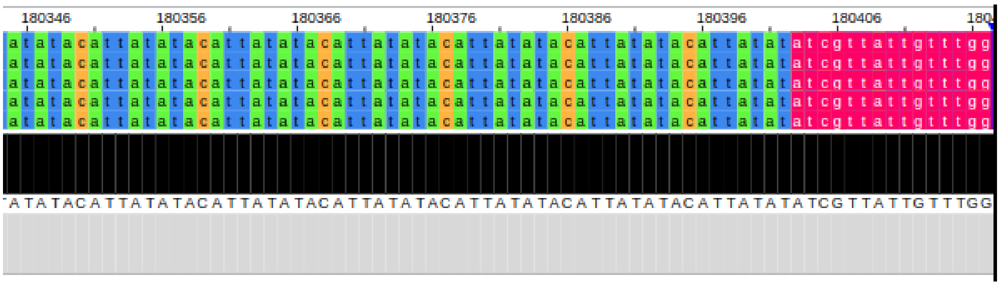

LCR4:

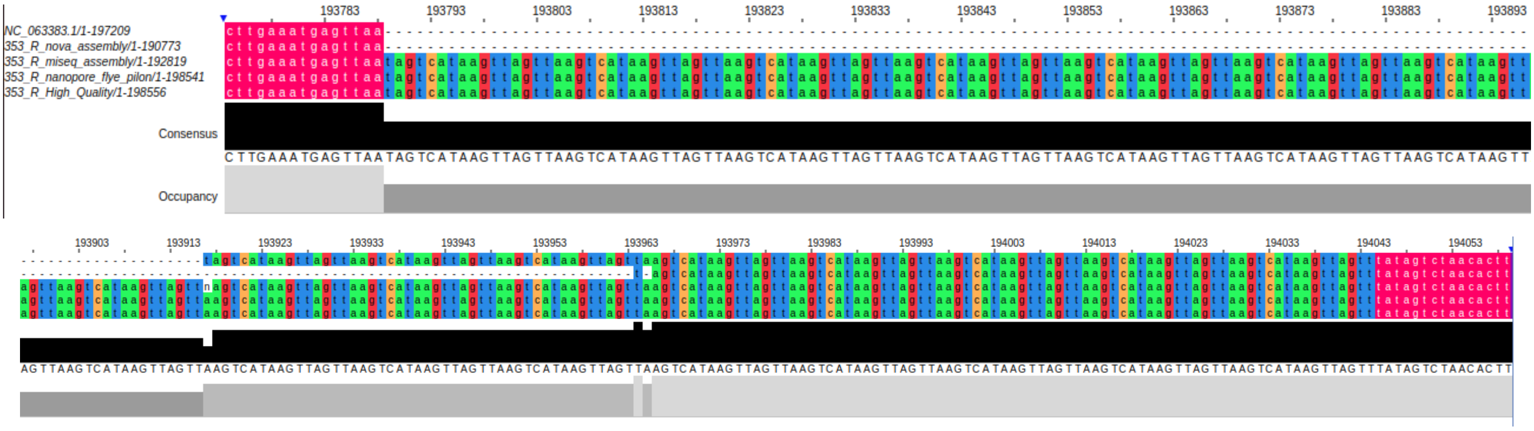

LCR5:

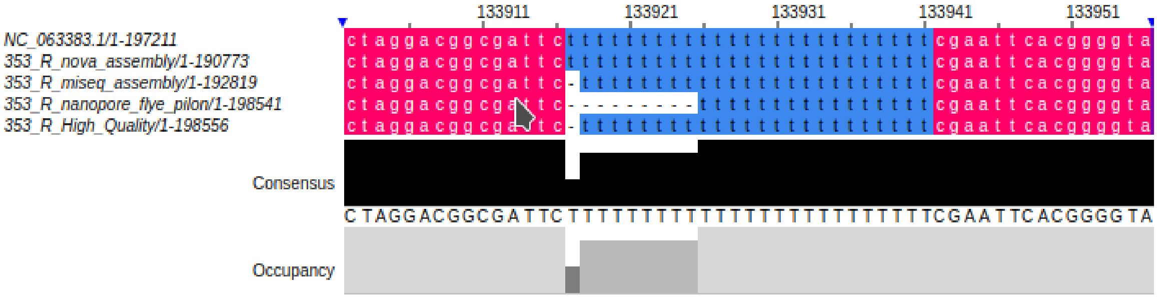

LCR6:

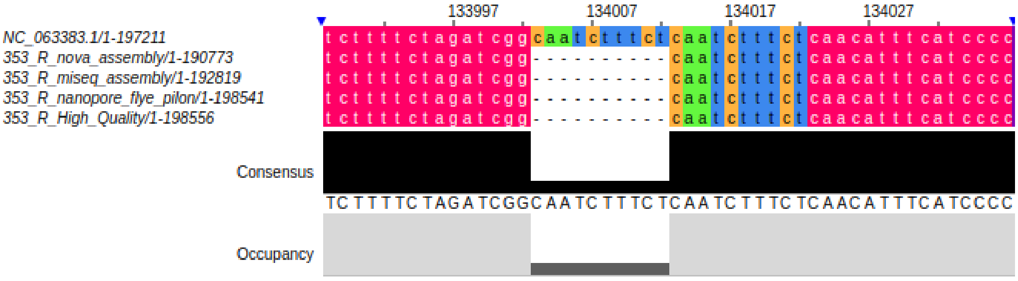

LCR7:

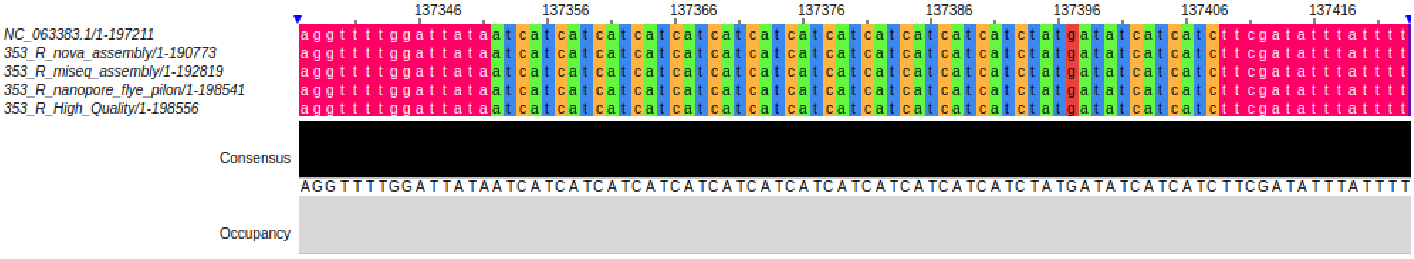

LCR8:

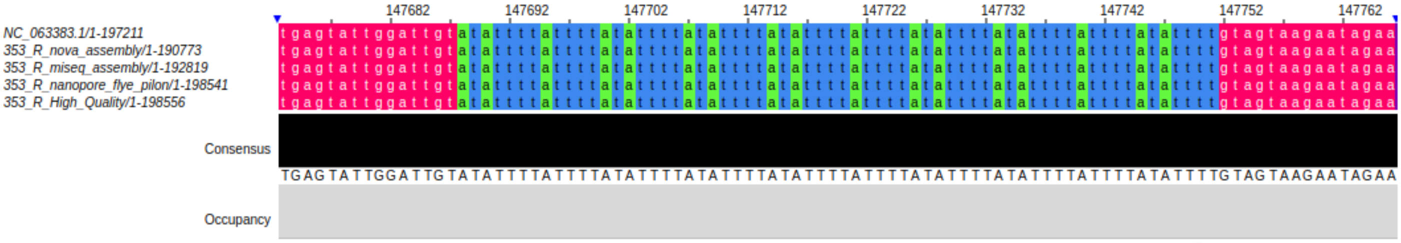

LCR9:

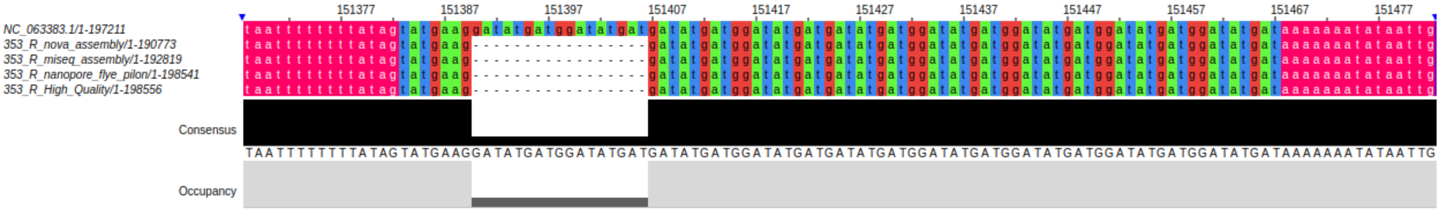

LCR10:

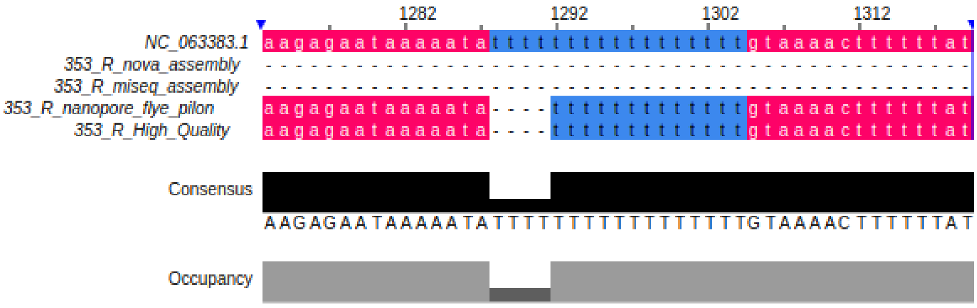

LCR11:

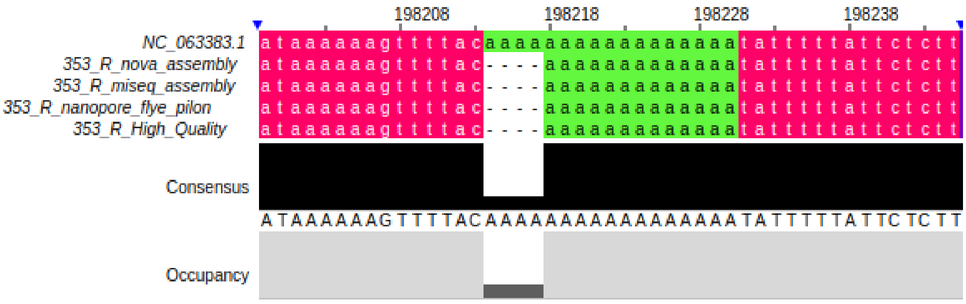

LCR12:

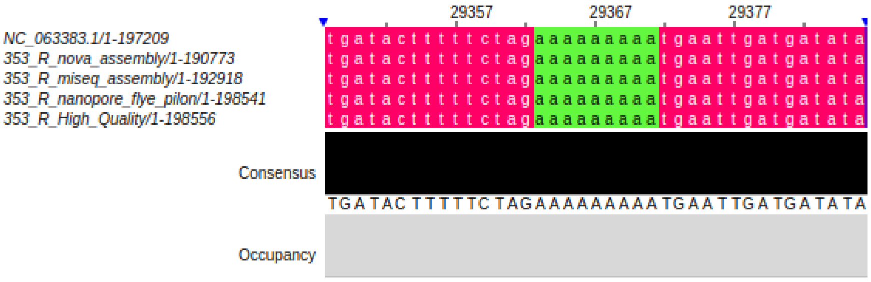

LCR13:

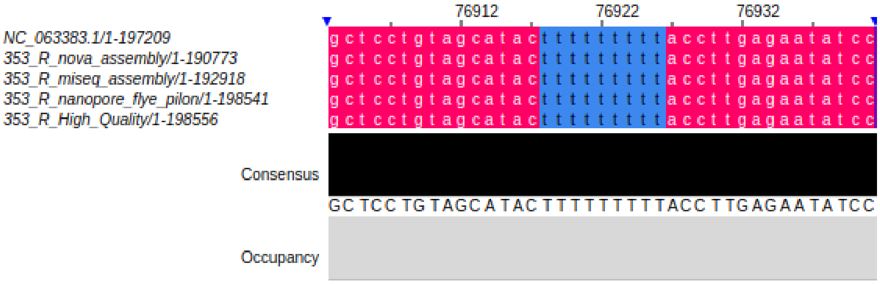

LCR14:

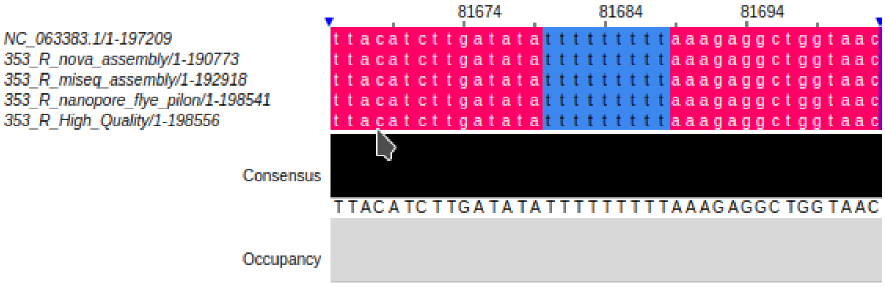

LCR15:

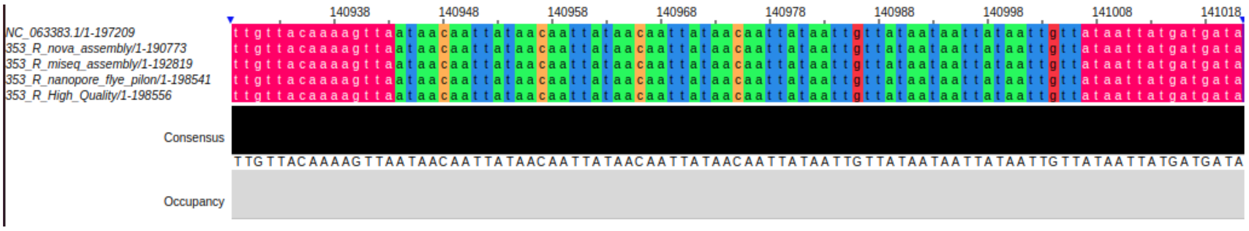

LCR16:

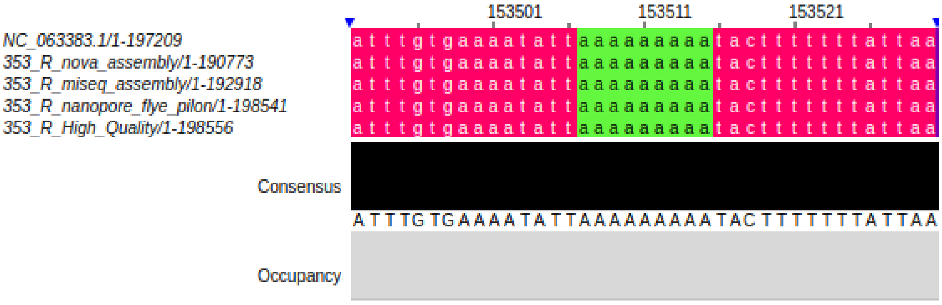

LCR17:

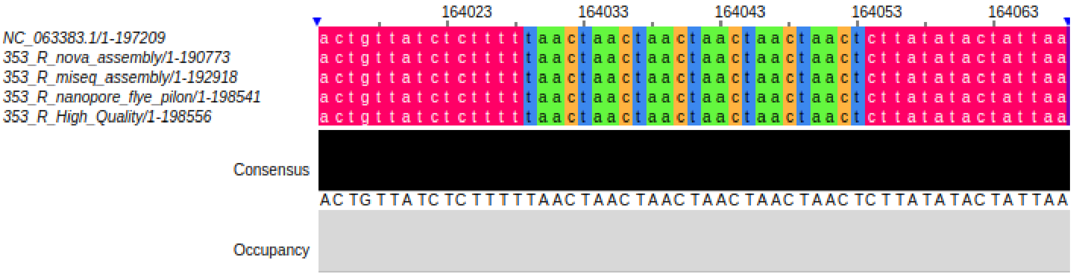

LCR18:

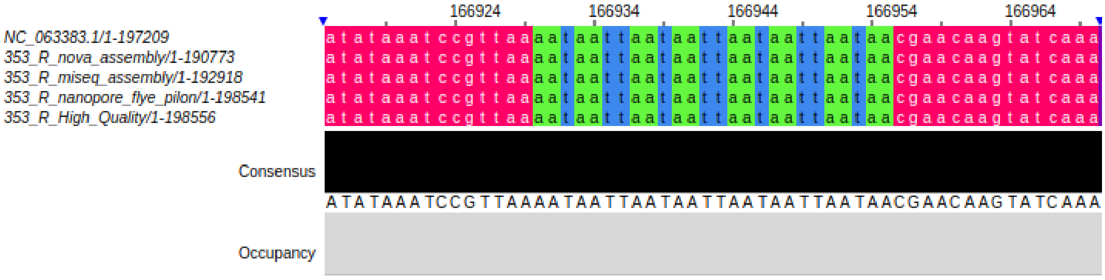

LCR19:

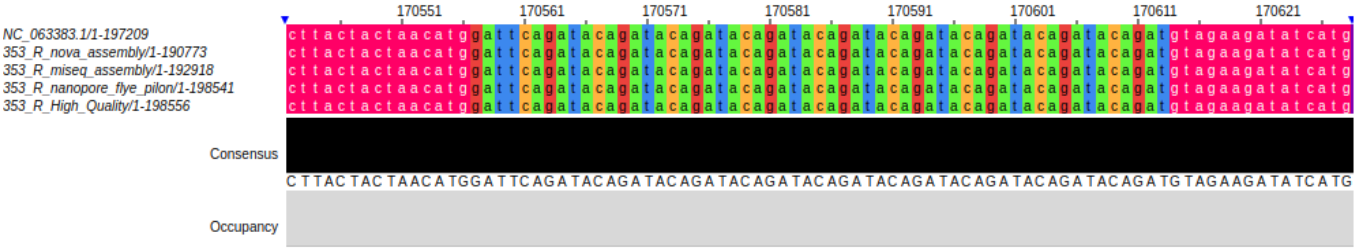

LCR20:

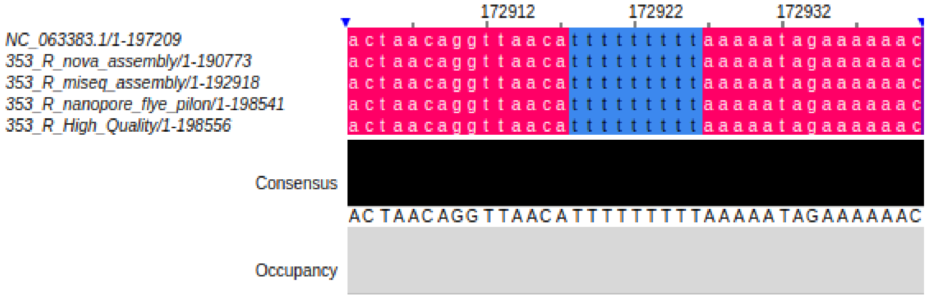

LCR21:

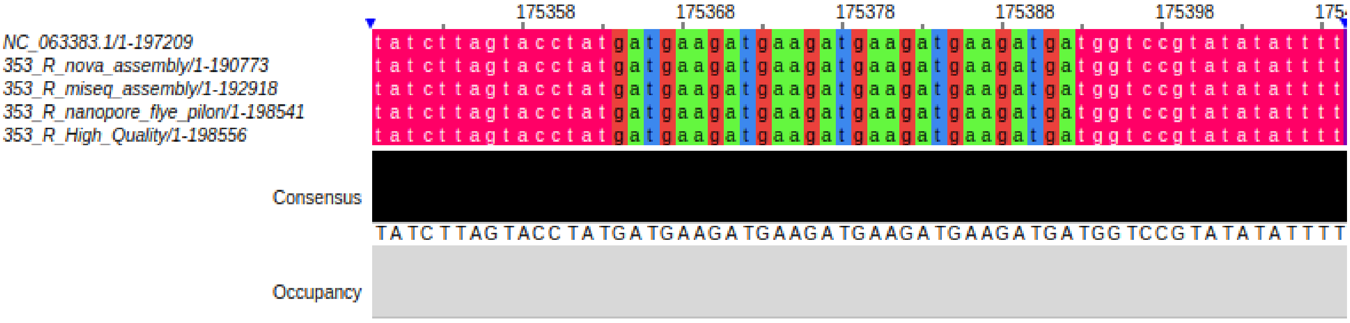

B. Nanopore samples analysis downloaded from SRA

Monkeypox samples with nanopore data available in SRA (August 10, 2022) were downloaded from NCBI webpage (https://www.ncbi.nlm.nih.gov/sra) resulting in 35 samples. Next, reads were de novo assembled using flye assembler v2.9-b1768 (Kolmogorov et al. 2019) with default parameters in nanopore raw reads mode, and analyzed using a modified version of strsearch (https://github.com/BU-ISCIII/MPXstreveal). Reads spanning the defined LCR3, LCR1 and LCR4 regions were identified. Those with both flanking reads with 0 mismatches were collected, aligned and generated a consensus sequence. Potential number of repeats of selected LCRs in each sample were inspected by comparing the assembled genome and the strsearch result according to **Supplementary data 6**.

The “de novo" assembly method only resulted in the resolution of a few complete genomes. To identify the number of LCRs repeats, we mostly used the method spanning the flanking regions. Although the results differ in some cases between bioinformatic methods, and we cannot validate the exact numbers of repeats experimentally since we do not have access to the samples, the values obtained show clear differences in the tendency of the number of repeats. For clarity, the most conservative number was selected, considering the biology of MPXV replication, the solution that was supported by the highest number of genomic data, and the selection of the solution that underestimated variation.
