# Supplementary material for: Monkeypox virus genomic accordion strategies
Source: Nat Commun. 2024 Apr 18;15:3059. doi: 10.1038/s41467-024-46949-7 (PMC11026394; doi:10.1038/s41467-024-46949-7)
Supplement: Supplementary file 3 — Description of Additional Supplementary Files [file 41467_2024_46949_MOESM3_ESM.pdf]

Description of Supplementary Data

File name: Supplementary Data 1. NovaSeq sequencing quality control values

File name: Supplementary Data 2. Low-complexity regions (LCRs) in the monkeypox virus (MPXV) high-quality genome (HQG) sequence 353R
Listed are annotated positions (according to the reference MPXV-M5312_HM12_Rivers isolate genome sequence), sequence, and flanking regions of each area, as described previously [114]. ID, identification; STR, short tandem repeats.

File name: Supplementary Data 3. Short tandem repeats (STRs) in low-complexity regions (LCRs)
Detailed information on STRs of the entire dataset used for analysis in **Figure 2**.

File name: Supplementary Data 4. Phylogenetic analysis
Detailed information on groups depicted in **Figure S2**. SNP, single-nucleotide polymorphism.

File name: Supplementary Data 5. Genome sequences used in the study
GI, genome identifier; NA, not applicable.

File name: Supplementary Data 6. Characterization and validation of non-randomly distributed low-complexity regions (LCRs) in the monkeypox virus (MPXV) 353R genome sequence
Detailed information on the represented materials, along with originator and epidemiological data, used for analysis in **Figure 2**. SRA, Sequence Read Archive (SRA); ID, identification; QC, quality control.

File name: Supplementary Data 7. Single-nucleotide polymorphism (SNPs) called against the subclade IIb lineage A reference isolate MPXV-M5312_HM12_Rivers genome sequence for subclade IIb lineage B.1 353R and MPXV_USA_2022_MA001 genome sequences.

File name: Supplementary Data 8. Top Early differentially expressed MPXV genes.
Wald test performed in DESEQ analysis. *p*-values were corrected by Benjamini–Hochberg (BH) adjustment, p-values indicated in last column.
