# Supplementary material for: Monkeypox virus genomic accordion strategies
Source: Nat Commun. 2024 Apr 18;15:3059. doi: 10.1038/s41467-024-46949-7 (PMC11026394; doi:10.1038/s41467-024-46949-7)
Supplement: Supplementary file 12 — Reporting Summary [file 41467_2024_46949_MOESM12_ESM.pdf]

nature portfolio

Corresponding author(s): Gustavo Palacios

Last updated by author(s): Mar 12, 2024

# Reporting Summary

Nature Portfolio wishes to improve the reproducibility of the work that we publish. This form provides structure for consistency and transparency in reporting. For further information on Nature Portfolio policies, see our Editorial Policies and the Editorial Policy Checklist.

## Statistics

For all statistical analyses, confirm that the following items are present in the figure legend, table legend, main text, or Methods section.

| n/a | Confirmed | |
|---|---|---|
| ☐ | ☒ | The exact sample size (*n*) for each experimental group/condition, given as a discrete number and unit of measurement |
| ☐ | ☒ | A statement on whether measurements were taken from distinct samples or whether the same sample was measured repeatedly |
| ☐ | ☒ | The statistical test(s) used AND whether they are one- or two-sided<br>*Only common tests should be described solely by name; describe more complex techniques in the Methods section.* |
| ☐ | ☒ | A description of all covariates tested |
| ☐ | ☒ | A description of any assumptions or corrections, such as tests of normality and adjustment for multiple comparisons |
| ☐ | ☒ | A full description of the statistical parameters including central tendency (e.g. means) or other basic estimates (e.g. regression coefficient) AND variation (e.g. standard deviation) or associated estimates of uncertainty (e.g. confidence intervals) |
| ☐ | ☒ | For null hypothesis testing, the test statistic (e.g. *F*, *t*, *r*) with confidence intervals, effect sizes, degrees of freedom and *P* value noted<br>*Give P values as exact values whenever suitable.* |
| ☒ | ☐ | For Bayesian analysis, information on the choice of priors and Markov chain Monte Carlo settings |
| ☐ | ☒ | For hierarchical and complex designs, identification of the appropriate level for tests and full reporting of outcomes |
| ☒ | ☐ | Estimates of effect sizes (e.g. Cohen's *d*, Pearson's *r*), indicating how they were calculated |

*Our web collection on statistics for biologists contains articles on many of the points above.*

## Software and code

Policy information about availability of computer code

Data collection

Study population
This study includes confirmed human mpox cases diagnosed from May 18 to July 14, 2022, at the Centro Nacional de Microbiología (CNM), Instituto de Salud Carlos III, Madrid, Spain. The study was performed as part of the public health response to the current mpox epidemic by the Spanish Ministry of Health. Sample information is listed in Supplementary Data 1 and Supplementary Data 5.
The samples used in this work were obtained in the context of the Microbiological Surveillance and Diagnosis Program for the mpox outbreak conducted by the Centro Nacional de Microbiología, Instituto de Salud Carlos III. The study was based on routine testing, did not involve any additional sampling or tests and stored RNA extracts were used, so specific ethical approval was not required for this study. All sequenced viruses corresponded to those to patients that gave consent to be analyzed for diagnosis or surveillance purposes.

Study sample processing
Swabs of vesicular lesions from study patients in viral transport media were sent refrigerated to CNM. Nucleic acids were extracted at CNM using either QIAamp MinElute Virus Spin (DNA) (#57704 )or QIAamp Viral RNA (#52904) Mini kits (Qiagen, Germantown, MD, USA) according to the manufacturer's recommendations. Inactivation of samples was conducted in a certified class II biological safety cabinet in a biosafety level (BSL) 2 laboratory using BSL-3 best practices with appropriate personal protective equipment.

Monkeypox virus (MPXV) laboratory confirmation
MPXV detection by PCR in a sample was considered laboratory confirmation and resulted in inclusion of the swab in the study. A previously described orthopoxvirus-generic real-time PCR (qPCR) was used for screening 71. A previously described conventional validated nested PCR targeting OPG002 (Cop-C22L) (encoding a TFN receptor) was used for results confirmation 72.

MPXV genome sequencing

Sequencing libraries were prepared with a tagmentation-based Illumina DNA Prep kit (#20060060 Illumina, San Diego, CA, USA) and run in a NovaSeq 6000 SP Reagent Kit (#20028312 Illumina) flow cell using 2x150 paired-end sequencing. To improve assembly quality, the library from swab 353R, an unpassaged vesicular fluid from a confirmed case, was also run in a MiSeq Reagent Kit v3 (#MS-102-3003 Illumina) flow cell using 2x300 paired-end sequencing. Additionally, sample 353R was also analyzed by single-molecule methods using Nanopore sequencing (Oxford Nanopore Technologies, Oxford, UK). For Nanopore sequencing, 210 ng of DNA was extracted from swab 353R and used to prepare a sequence library with a Rapid Sequencing Kit (#SQK-RAD114 Oxford Nanopore Technologies); the library was analyzed in an FLO-MIN106D (#FLO-MIN106D Oxford Nanopore Technologies) flow cell for 25 h. The process rendered 1.12 Gb of filter-passed bases.

Data analysis

All scripts and codes used for this study can be found at github repository: (https://github.com/BU-ISCIII/MPXstreveal). https://doi.org/10.5281/zenodo.10721675

De novo assembly and annotation of subclade IIb lineage B.1 MPXV genome sequence 353R

Due to the high yield of MPXV genomic material in a preparatory run, swab 353R was selected as source material for the determination of an MPXV high-quality genome (HQG) sequence. Single-molecule long-sequencing reads were preprocessed using Porechop v0.3.2pre 73 with default parameters. Reads were de novo assembled using Flye v2.9-b1768 74 in single-molecule sequencing raw read mode with default parameters, resulting in one MPXV contig of 198,254 bp. Short 2x150 sequencing reads were mapped with Bowtie2 v2.4.4 75 against the selected contig, and resulting BAM files were used to correct the assembly using Pilon v1.24 76. At this intermediate step, this corrected sequence was used as a reference in the nf-core/viralrecon v2.4.1 pipeline 77 for mapping and consensus generation with short-read sequencing. The allele frequency threshold of 0.5 was used for including variant positions in the corrected contig.

Short MiSeq 2x300 and NovaSeq 2x150 sequencing reads were also assembled de novo using the nf-core/viralrecon v2.4.1 pipeline, written in Nextflow 78 in collaboration between the nf-core community 79 and the Unidad de Bioinformática, Instituto de Salud Carlos III, Madrid, Spain (https://github.com/BU-ISCIII). FASTQ files containing raw reads were quality controlled using FASTQC v0.11.9 80. Raw reads were trimmed using fastp v0.23.2 81. The sliding-window quality-filtering approach was performed, scanning the read with a 4-base-wide sliding window and cutting 3' and 5' base ends when average quality per base dropped below a Qphred33 of 20. Reads shorter than 50 bp and reads with more than 10% read quality under Qphred 20 were removed. Host genome reads were removed via kmer-based mapping of the trimmed reads against the human genome reference sequence GRCh38 (https://www.ncbi.nlm.nih.gov/data-hub/genome/GCF_000001405.26/) using Kraken 2 v2.1.2 82. The remaining non-host reads were assembled using SPADES v3.15.3 83,84 in rnaviral mode. A fully ordered MPXV genome sequence was generated using ABACAS v1.3.1 85, based on the MPXV isolate MPXV_USA_2022_MA001 (Nextstrain subclade IIb lineage B.1) sequence (GenBank #ON563414.3) 86. The independently obtained de novo assemblies and reference-based consensus genomes obtained from swab 353R were aligned using MAFFT v7.475 87 and visually inspected for variation using Jalview v2.11.0 88.

Systematic identification of low-complexity regions in orthopoxvirus genomes

Detection of STRs in the HQG sequence and other orthopoxvirus genomes was performed with Tandem repeats finder 89, using default parameters. Briefly, the algorithm works without the need to specify either the pattern or its length. Tandem repeats are identified considering percent identity and frequency of insertion (ins) or deletion (del) of bases (indels) between adjacent pattern copies, using statistically based recognition criteria. Since Tandem repeats finder does not detect single-nucleotide repeats, we developed an R script to systematically identify homopolymers of at least 9 nucleotide residues in all available orthopoxvirus genome sequences. STRs and homopolymers were annotated as low-complexity regions (LCRs).

Curation of low-complexity regions in the MPXV high-quality virus genome sequence

We curated LCRs in the HQG sequence using a modified version of STRsearch 90. Once provided with identifying flanking regions, STRsearch performed a profile analysis of STRs in massively parallel sequencing data. To ensure high-quality characterization of LCR alleles, we modified the script (https://github.com/BU-ISCIII/MPXstreveal) to complement reverse reads that map against the reverse genome strand according to their BAM flags. In addition, output was modified to add information later accessed by a custom Python script to select only reads containing both LCR flanking regions. All LCRs in the HQG sequence were manually validated using STRsearch results and de novo assemblies obtained from all sequencing approaches. When an LCR was only resolved by single-molecule long-sequencing technologies (LCR pair 1/4 and LCR3), we also analyzed publicly available data by downloading all single-molecule long-sequencing data from the National Center for Biotechnology Information (NCBI) Sequence Read Archive (SRA) (https://www.ncbi.nlm.nih.gov/sra) as of August 10, 2022, and analyzed the data according to Supplementary Note 1.

Final MPXV high-quality virus genome sequence assembly

The consensus genome constructed with the nf-core/viralrecon v2.4.1 pipeline using the corrected de novo contig as stated above, along with the resulting curated and validated consensus LCRs, were used to build the final HQG reference sequence using a custom Python script. The resulting HQG is available from the European Nucleotide Archive (#OX044336.2).

Generation of MPXV high-quality virus genome reference-based consensus sequence for all other samples

For the remaining specimens, sequencing reads were analyzed for MPXV genome sequence determination using the nf-core/viralrecon v2.4.1 pipeline. Trimmed reads were mapped with Bowtie2 v2.4.4 against the HQG sequence and the sequence of subclade IIb lineage A MPXV isolate M5312_HM12_Rivers (GenBank #MT903340.1) 91. Picard v2.26.10 92 and SAMtools v1.14 93 were used to generate MPXV genome mapping statistics. iVar v1.3.1 94, which calls for low-frequency and high-frequency variants, was used for variant calling. Variants with an allele frequency higher than 75% were kept to be included in the consensus genome sequence. BCFtools v1.14 95 was used to obtain the MPXV genome sequence consensus with filtered variants and masked genomic regions with coverage values lower than 10X. All variants, included or not, in the consensus genome sequence, were annotated using SnpEff v5.0e 96, and SnpSift v4.3 97. Final summary reports were created using MultiQC v1.11 98. Consensus genome sequences were analyzed with Nextclade v2.4.1 99 using the “MPXV (All clades)” dataset (timestamp 2022-08-19T12:00:00Z). Raw reads and consensus genomes are available from the European Nucleotide Archive (#ERS12168855–ERS12168865, #ERS12168867, #ERS12168868, #ERS13490510–ERS13490543).

Intra-host and inter-host allele frequency analyses

Intra-host genetic entropy (defined as -sum(Xi*log(Xi)), in which Xi denotes each of the allele frequencies in a position) was calculated according to the SNP frequencies of each position along the genome using nf-core/viralrecon v2.4.1 pipeline results. Similarly, genetic entropy for each LCR was calculated considering the frequencies of repeat lengths. LCR intra-host and inter-host variations in the sample set were analyzed using the modified version of STRsearch. As a filter for quality for this analysis, STRsearch results (Supplementary Data 5) were filtered, keeping alleles with at least 10 reads spanning the region and allele frequency above 0.03. Quality control and allele frequency graphs were created using a customized R script. Pairwise genetic distances between samples were calculated as Euclidean distances (defined as /X-Y/=sqrt(sum(xi-yi)^2), in which xi and yi are the allele frequencies of sample X and Y at a given position, respectively), thus accounting for the major and minor alleles at each analyzed position. Distances were calculated individually for each variable LCR (STRs 2, 5, 7, 10, 11, and 21) and for each of all 5,422 SNPs showing inter-sample variability (compared to MPXV-M5312_HM12_Rivers). The distributions of inter-sample distances were compared between LCRs using a Kruskal–Wallis test ($\chi2$ p-values) followed by multiple pairwise-comparison between groups

(Wilcoxon test), with p-values subjected to the false discovery rate (FDR) correction. A randomization test was used to test whether inter-sample variability in LCRs is higher than that in SNPs: first, the average Euclidean distance for each LCR and each SNP position was calculated; then, the average value of each LCR was compared to a random sample of 1,000 values from the distribution of mean distances from the SNPs along the genome. The p-value was calculated from the percentage of times that the mean of the LCR was higher than the randomly taken values from the SNPs.

Phylogenetic analysis of the MPXV central conserved region
Variant calling and SNP matrix generation was performed using Snippy v4.4.5 100, including sequence samples and representative MPXV genome sequences downloaded from GenBank (Supplementary Data 5). The SNP matrix with both invariant and variant sites was used for phylogenetic analysis using IQ-Tree 2 v. 2.1.4-beta 101 via predicted model K3Pu+F+I and 1,000 bootstrap replicates. A phylogenetic tree was visualized and annotated using iTOL v6.5.8 102. The SNP matrix was also used for generating the haplotype network using PopArt v1.7 103.

Selected MPXV ORF analysis
Representative orthopoxvirus genomes 32 were downloaded from GenBank together with the consensus genome sequences from the specimens analyzed in this study (Supplementary Data 5). MPXV genomes were assigned to clades and lineages according to the most recent nomenclature recommendations according to Nextstrain 14 using Nextclade v2.4.1. Annotations from RefSeq #NC_063383.1 (subclade IIb lineage A MPXV virus isolate MPXV-M5312_HM12_Rivers) GFF file were transferred to all FASTA genome sequences using Liftoff v1.6.3 104. OPG153 was extracted using AGAT v0.9.1 105 and multi-FASTA files were generated for each group and gene. OPG204 and OPG208 alternative annotation start site ORFs were re-annotated in Geneious Prime (Biomatters, San Diego, CA, USA), and extracted as new alignments. We used MUSCLE v3.8.1551 for aligning each multi-FASTA file and Jalview v2.11.0 for inspecting and editing the alignments. Finally, MetaLogo v1.1.2 106 was used for creating and aligning the sequence logos for each orthopoxvirus group of the OPG153/LCR7, OPG204/LCR21, and OPG208/LCR3 areas.

Comparison of LCR frequencies in protein functional groups
The potential biological impact of LCRs was evaluated by mapping the frequency and location of STRs and homopolymers in the orthopoxvirus genome and considering the biological function of the affected genes. The frequency of inclusion of LCRs between distinct functional groups of genes was compared as previously described 32. Orthopoxvirus genomes (n=231, Akhmeta virus [AKMV]: n=6 sequences; alaskapox virus [AKPV]: n=1; cowpox virus [CPXV]: n=82; ectromelia virus [ECTV]: n=5; MPXV: n=62; VACV: n=18; VARV: n=57) include 216 functionally annotated OPGs classified in 6 categories ("Housekeeping genes/Core" ANK/PRANC family, Bcl-2 domain family, BTB/Kelch domain family, PIE family, and "Accessory/Other" [e.g., virus–host interacting genes]). The frequency was calculated after normalizing the number of LCRs registered with the sample size of the OPG alignment in the categories described above. Statistical analysis of the significance of differences was performed by applying a Kruskal–Wallis test ($\chi 2$ p-values) followed by a non-parametric multiple pairwise comparison between groups (Wilcoxon test), with p-values subjected to FDR correction.

Data selection
RNA-seq samples were retrieved from NCBI SRA (ncbi.nlm.nih.gov/sra) under accession ERP141806. The RNA-seq data are from MPXV isolated from skin lesion samples from a patient; samples had been cultured in CV-1 cells for 1–24 hours post-infection, 3 replicates each. The original samples were subjected to total RNA isolation, library preparation, poly(A)+ enrichment, and RNA sequencing with ONT MinIon. The BAM files retrieved from SRA were preprocessed files from which reads have been aligned to MPXV ON563414.3 and human GCF_015252025.1 43.

DEG analyses
Default parameters were set in htseq-count 107, which was used to obtain transcript counts for each MPXV gene (ON563414.3 reference) for every sample. Differential gene-expression analyses were then performed with DESeq2 (R package) 108 to differentiate early (those overexpressed at early time points) versus late (most expressed at late time points) viral genes. A linear-regression analysis considering the variables "replicate" and "timepoint" was performed.

Definition of promoters
For the top 10 early and late genes (defined as those genes with padj <0.05 that had the largest absolute log2 fold change values), we retrieved the sequence representing the 100 nucleotides upstream of their canonical start codon (ATG), considering the coordinates of the reference genome ON563414.3. Early and late genes were then aligned separately with the Muscle alignment tool 109 from MEGA11 110. VACV early and late genes have unique conserved TATA boxes 111-113. We used these reports as templates to define the likely promoters in MPXV. After defining the more likely consensus early and late promoters from the general viral transcripts, we identified the specific promoters of OPG204 (which was among the top upregulated genes at 1 hour post-infection) and of OPG208 (not overexpressed at 1 nor at 24 hours post-infection but expressed at the earliest timepoint).

Read depth analysis
We later analyzed the region spanning 40 bases before the start of translation to the first 10 nucleotides from the analyzed coding region of all top early genes using SAMtools 93 to assess the read depth at the 5' end, and later we compared them to OPG204 and OPG208, which were both defined as early genes. The idea behind this analysis is that, given poly(A)+ enrichment, regions that are transcribed (e.g., containing a coding region and untranslated 5' region) should be covered at significantly higher depth than non-transcribed areas (noise).

For manuscripts utilizing custom algorithms or software that are central to the research but not yet described in published literature, software must be made available to editors and reviewers. We strongly encourage code deposition in a community repository (e.g. GitHub). See the Nature Portfolio guidelines for submitting code & software for further information.

# Data

Policy information about availability of data

All manuscripts must include a data availability statement. This statement should provide the following information, where applicable:

- Accession codes, unique identifiers, or web links for publicly available datasets
- A description of any restrictions on data availability
- For clinical datasets or third party data, please ensure that the statement adheres to our policy

The data generated in this study are provided within the article, Supplementary Information, and Supplementary Data. MPXV High-quality genome sequence is

available in the European Nucleotide Archive (ENA) database (Accession number OX044336) https://www.ebi.ac.uk/ena/browser/view/OX044336, accession numbers for all the samples sequenced in this manuscript can be found in Supplementary Data 1. Further information and requests for resources and reagents should be directed to and will be fulfilled by the lead contact, Gustavo Palacios (gustavo.palacios@mssm.edu).

## Research involving human participants, their data, or biological material

Policy information about studies with human participants or human data. See also policy information about sex, gender (identity/presentation), and sexual orientation and race, ethnicity and racism.

| | |
|---|---|
| Reporting on sex and gender | We do not report any information on sex and gender |
| Reporting on race, ethnicity, or other socially relevant groupings | We do not report any information regarding race, ethnicity or other socially relevant groupings |
| Population characteristics | Viral smaples were extracted from patients on the context of Microbiological Surveillance and Diagnosis Program for the mpox outbreak. |
| Recruitment | The samples used in this work were obtained in the context of the Microbiological Surveillance and Diagnosis Program for the mpox outbreak conducted by the Centro Nacional de Microbiología, Instituto de Salud Carlos III. The study was based on routine testing, did not involve any additional sampling or tests and stored RNA extracts were used, so specific ethical approval was not required for this study. All sequenced viruses corresponded to those to patients that gave consent to be analyzed for diagnosis or surveillance purposes. |
| Ethics oversight | Specifical ethical approval was not required for this study |

Note that full information on the approval of the study protocol must also be provided in the manuscript.

# Field-specific reporting

Please select the one below that is the best fit for your research. If you are not sure, read the appropriate sections before making your selection.

☒ Life sciences ☐ Behavioural & social sciences ☐ Ecological, evolutionary & environmental sciences

For a reference copy of the document with all sections, see nature.com/documents/nr-reporting-summary-flat.pdf

# Life sciences study design

All studies must disclose on these points even when the disclosure is negative.

| | |
|---|---|
| Sample size | We report sample size in all figure legends in the manuscript. Sample size is usually derived from what can be found in public respositoires and the sequencing quality of consensus and raw data associated to those sequences |
| Data exclusions | No data was excluded |
| Replication | Code and data availability ensures our experimental findings are reproducible |
| Randomization | We do not perform any animal experiment that requires randomization |
| Blinding | Blinding was not relevant to the study since we are not addressing any differences between groups |

# Behavioural & social sciences study design

All studies must disclose on these points even when the disclosure is negative.

| | |
|---|---|
| Study description | *Briefly describe the study type including whether data are quantitative, qualitative, or mixed-methods (e.g. qualitative cross-sectional, quantitative experimental, mixed-methods case study).* |
| Research sample | *State the research sample (e.g. Harvard university undergraduates, villagers in rural India) and provide relevant demographic information (e.g. age, sex) and indicate whether the sample is representative. Provide a rationale for the study sample chosen. For studies involving existing datasets, please describe the dataset and source.* |
| Sampling strategy | *Describe the sampling procedure (e.g. random, snowball, stratified, convenience). Describe the statistical methods that were used to predetermine sample size OR if no sample-size calculation was performed, describe how sample sizes were chosen and provide a rationale for why these sample sizes are sufficient. For qualitative data, please indicate whether data saturation was considered, and what criteria were used to decide that no further sampling was needed.* |
| Data collection | *Provide details about the data collection procedure, including the instruments or devices used to record the data (e.g. pen and paper,* |

| | |
|---|---|
| Data collection | *computer, eye tracker, video or audio equipment) whether anyone was present besides the participant(s) and the researcher, and whether the researcher was blind to experimental condition and/or the study hypothesis during data collection.* |
| Timing | *Indicate the start and stop dates of data collection. If there is a gap between collection periods, state the dates for each sample cohort.* |
| Data exclusions | *If no data were excluded from the analyses, state so OR if data were excluded, provide the exact number of exclusions and the rationale behind them, indicating whether exclusion criteria were pre-established.* |
| Non-participation | *State how many participants dropped out/declined participation and the reason(s) given OR provide response rate OR state that no participants dropped out/declined participation.* |
| Randomization | *If participants were not allocated into experimental groups, state so OR describe how participants were allocated to groups, and if allocation was not random, describe how covariates were controlled.* |

# Ecological, evolutionary & environmental sciences study design

All studies must disclose on these points even when the disclosure is negative.

| | |
|---|---|
| Study description | *Briefly describe the study. For quantitative data include treatment factors and interactions, design structure (e.g. factorial, nested, hierarchical), nature and number of experimental units and replicates.* |
| Research sample | *Describe the research sample (e.g. a group of tagged Passer domesticus, all Stenocereus thurberi within Organ Pipe Cactus National Monument), and provide a rationale for the sample choice. When relevant, describe the organism taxa, source, sex, age range and any manipulations. State what population the sample is meant to represent when applicable. For studies involving existing datasets, describe the data and its source.* |
| Sampling strategy | *Note the sampling procedure. Describe the statistical methods that were used to predetermine sample size OR if no sample-size calculation was performed, describe how sample sizes were chosen and provide a rationale for why these sample sizes are sufficient.* |
| Data collection | *Describe the data collection procedure, including who recorded the data and how.* |
| Timing and spatial scale | *Indicate the start and stop dates of data collection, noting the frequency and periodicity of sampling and providing a rationale for these choices. If there is a gap between collection periods, state the dates for each sample cohort. Specify the spatial scale from which the data are taken* |
| Data exclusions | *If no data were excluded from the analyses, state so OR if data were excluded, describe the exclusions and the rationale behind them, indicating whether exclusion criteria were pre-established.* |
| Reproducibility | *Describe the measures taken to verify the reproducibility of experimental findings. For each experiment, note whether any attempts to repeat the experiment failed OR state that all attempts to repeat the experiment were successful.* |
| Randomization | *Describe how samples/organisms/participants were allocated into groups. If allocation was not random, describe how covariates were controlled. If this is not relevant to your study, explain why.* |
| Blinding | *Describe the extent of blinding used during data acquisition and analysis. If blinding was not possible, describe why OR explain why blinding was not relevant to your study.* |

Did the study involve field work? ☐ Yes ☐ No

## Field work, collection and transport

| | |
|---|---|
| Field conditions | *Describe the study conditions for field work, providing relevant parameters (e.g. temperature, rainfall).* |
| Location | *State the location of the sampling or experiment, providing relevant parameters (e.g. latitude and longitude, elevation, water depth).* |
| Access & import/export | *Describe the efforts you have made to access habitats and to collect and import/export your samples in a responsible manner and in compliance with local, national and international laws, noting any permits that were obtained (give the name of the issuing authority, the date of issue, and any identifying information).* |
| Disturbance | *Describe any disturbance caused by the study and how it was minimized.* |

# Reporting for specific materials, systems and methods

We require information from authors about some types of materials, experimental systems and methods used in many studies. Here, indicate whether each material, system or method listed is relevant to your study. If you are not sure if a list item applies to your research, read the appropriate section before selecting a response.

## Materials & experimental systems

| n/a | Involved in the study |
|---|---|
| ☒ | ☐ Antibodies |
| ☒ | ☐ Eukaryotic cell lines |
| ☒ | ☐ Palaeontology and archaeology |
| ☒ | ☐ Animals and other organisms |
| ☒ | ☐ Clinical data |
| ☒ | ☐ Dual use research of concern |
| ☒ | ☐ Plants |

## Methods

| n/a | Involved in the study |
|---|---|
| ☒ | ☐ ChIP-seq |
| ☒ | ☐ Flow cytometry |
| ☒ | ☐ MRI-based neuroimaging |

# Antibodies

Antibodies used — *Describe all antibodies used in the study; as applicable, provide supplier name, catalog number, clone name, and lot number.*

Validation — *Describe the validation of each primary antibody for the species and application, noting any validation statements on the manufacturer's website, relevant citations, antibody profiles in online databases, or data provided in the manuscript.*

# Eukaryotic cell lines

Policy information about cell lines and Sex and Gender in Research

Cell line source(s) — *State the source of each cell line used and the sex of all primary cell lines and cells derived from human participants or vertebrate models.*

Authentication — *Describe the authentication procedures for each cell line used OR declare that none of the cell lines used were authenticated.*

Mycoplasma contamination — *Confirm that all cell lines tested negative for mycoplasma contamination OR describe the results of the testing for mycoplasma contamination OR declare that the cell lines were not tested for mycoplasma contamination.*

Commonly misidentified lines (See ICLAC register) — *Name any commonly misidentified cell lines used in the study and provide a rationale for their use.*

# Palaeontology and Archaeology

Specimen provenance — *Provide provenance information for specimens and describe permits that were obtained for the work (including the name of the issuing authority, the date of issue, and any identifying information). Permits should encompass collection and, where applicable, export.*

Specimen deposition — *Indicate where the specimens have been deposited to permit free access by other researchers.*

Dating methods — *If new dates are provided, describe how they were obtained (e.g. collection, storage, sample pretreatment and measurement), where they were obtained (i.e. lab name), the calibration program and the protocol for quality assurance OR state that no new dates are provided.*

☐ Tick this box to confirm that the raw and calibrated dates are available in the paper or in Supplementary Information.

Ethics oversight — *Identify the organization(s) that approved or provided guidance on the study protocol, OR state that no ethical approval or guidance was required and explain why not.*

Note that full information on the approval of the study protocol must also be provided in the manuscript.

# Animals and other research organisms

Policy information about studies involving animals; ARRIVE guidelines recommended for reporting animal research, and Sex and Gender in Research

Laboratory animals — *For laboratory animals, report species, strain and age OR state that the study did not involve laboratory animals.*

Wild animals — *Provide details on animals observed in or captured in the field; report species and age where possible. Describe how animals were caught and transported and what happened to captive animals after the study (if killed, explain why and describe method; if released, say where and when) OR state that the study did not involve wild animals.*

Reporting on sex — *Indicate if findings apply to only one sex; describe whether sex was considered in study design, methods used for assigning sex. Provide data disaggregated for sex where this information has been collected in the source data as appropriate; provide overall*


April 2023

*numbers in this Reporting Summary. Please state if this information has not been collected. Report sex-based analyses where performed, justify reasons for lack of sex-based analysis.*

| | |
|---|---|
| Field-collected samples | *For laboratory work with field-collected samples, describe all relevant parameters such as housing, maintenance, temperature, photoperiod and end-of-experiment protocol OR state that the study did not involve samples collected from the field.* |
| Ethics oversight | *Identify the organization(s) that approved or provided guidance on the study protocol, OR state that no ethical approval or guidance was required and explain why not.* |

Note that full information on the approval of the study protocol must also be provided in the manuscript.

# Clinical data

Policy information about clinical studies

All manuscripts should comply with the ICMJE guidelines for publication of clinical research and a completed CONSORT checklist must be included with all submissions.

| | |
|---|---|
| Clinical trial registration | *Provide the trial registration number from ClinicalTrials.gov or an equivalent agency.* |
| Study protocol | *Note where the full trial protocol can be accessed OR if not available, explain why.* |
| Data collection | *Describe the settings and locales of data collection, noting the time periods of recruitment and data collection.* |
| Outcomes | *Describe how you pre-defined primary and secondary outcome measures and how you assessed these measures.* |

# Dual use research of concern

Policy information about dual use research of concern

## Hazards

Could the accidental, deliberate or reckless misuse of agents or technologies generated in the work, or the application of information presented in the manuscript, pose a threat to:

| No | Yes | |
|---|---|---|
| ☒ | ☐ | Public health |
| ☒ | ☐ | National security |
| ☒ | ☐ | Crops and/or livestock |
| ☒ | ☐ | Ecosystems |
| ☒ | ☐ | Any other significant area |

## Experiments of concern

Does the work involve any of these experiments of concern:

| No | Yes | |
|---|---|---|
| ☒ | ☐ | Demonstrate how to render a vaccine ineffective |
| ☒ | ☐ | Confer resistance to therapeutically useful antibiotics or antiviral agents |
| ☒ | ☐ | Enhance the virulence of a pathogen or render a nonpathogen virulent |
| ☒ | ☐ | Increase transmissibility of a pathogen |
| ☒ | ☐ | Alter the host range of a pathogen |
| ☒ | ☐ | Enable evasion of diagnostic/detection modalities |
| ☒ | ☐ | Enable the weaponization of a biological agent or toxin |
| ☒ | ☐ | Any other potentially harmful combination of experiments and agents |

# Plants

Seed stocks

*Report on the source of all seed stocks or other plant material used. If applicable, state the seed stock centre and catalogue number. If plant specimens were collected from the field, describe the collection location, date and sampling procedures.*

Novel plant genotypes

*Describe the methods by which all novel plant genotypes were produced. This includes those generated by transgenic approaches, gene editing, chemical/radiation-based mutagenesis and hybridization. For transgenic lines, describe the transformation method, the number of independent lines analyzed and the generation upon which experiments were performed. For gene-edited lines, describe the editor used, the endogenous sequence targeted for editing, the targeting guide RNA sequence (if applicable) and how the editor was applied.*

Authentication

*Describe any authentication procedures for each seed stock used or novel genotype generated. Describe any experiments used to assess the effect of a mutation and, where applicable, how potential secondary effects (e.g. second site T-DNA insertions, mosiacism, off-target gene editing) were examined.*

# ChIP-seq

## Data deposition

☐ Confirm that both raw and final processed data have been deposited in a public database such as GEO.

☐ Confirm that you have deposited or provided access to graph files (e.g. BED files) for the called peaks.

Data access links
*May remain private before publication.*

*For "Initial submission" or "Revised version" documents, provide reviewer access links. For your "Final submission" document, provide a link to the deposited data.*

Files in database submission

*Provide a list of all files available in the database submission.*

Genome browser session
(e.g. UCSC)

*Provide a link to an anonymized genome browser session for "Initial submission" and "Revised version" documents only, to enable peer review. Write "no longer applicable" for "Final submission" documents.*

## Methodology

Replicates

*Describe the experimental replicates, specifying number, type and replicate agreement.*

Sequencing depth

*Describe the sequencing depth for each experiment, providing the total number of reads, uniquely mapped reads, length of reads and whether they were paired- or single-end.*

Antibodies

*Describe the antibodies used for the ChIP-seq experiments; as applicable, provide supplier name, catalog number, clone name, and lot number.*

Peak calling parameters

*Specify the command line program and parameters used for read mapping and peak calling, including the ChIP, control and index files used.*

Data quality

*Describe the methods used to ensure data quality in full detail, including how many peaks are at FDR 5% and above 5-fold enrichment.*

Software

*Describe the software used to collect and analyze the ChIP-seq data. For custom code that has been deposited into a community repository, provide accession details.*

# Flow Cytometry

## Plots

Confirm that:

☐ The axis labels state the marker and fluorochrome used (e.g. CD4-FITC).

☐ The axis scales are clearly visible. Include numbers along axes only for bottom left plot of group (a 'group' is an analysis of identical markers).

☐ All plots are contour plots with outliers or pseudocolor plots.

☐ A numerical value for number of cells or percentage (with statistics) is provided.

## Methodology

Sample preparation

*Describe the sample preparation, detailing the biological source of the cells and any tissue processing steps used.*

Instrument

*Identify the instrument used for data collection, specifying make and model number.*

Software

*Describe the software used to collect and analyze the flow cytometry data. For custom code that has been deposited into a community repository, provide accession details.*

Cell population abundance | *Describe the abundance of the relevant cell populations within post-sort fractions, providing details on the purity of the samples and how it was determined.*

Gating strategy | *Describe the gating strategy used for all relevant experiments, specifying the preliminary FSC/SSC gates of the starting cell population, indicating where boundaries between "positive" and "negative" staining cell populations are defined.*

☐ Tick this box to confirm that a figure exemplifying the gating strategy is provided in the Supplementary Information.

# Magnetic resonance imaging

## Experimental design

Design type | *Indicate task or resting state; event-related or block design.*

Design specifications | *Specify the number of blocks, trials or experimental units per session and/or subject, and specify the length of each trial or block (if trials are blocked) and interval between trials.*

Behavioral performance measures | *State number and/or type of variables recorded (e.g. correct button press, response time) and what statistics were used to establish that the subjects were performing the task as expected (e.g. mean, range, and/or standard deviation across subjects).*

## Acquisition

Imaging type(s) | *Specify: functional, structural, diffusion, perfusion.*

Field strength | *Specify in Tesla*

Sequence & imaging parameters | *Specify the pulse sequence type (gradient echo, spin echo, etc.), imaging type (EPI, spiral, etc.), field of view, matrix size, slice thickness, orientation and TE/TR/flip angle.*

Area of acquisition | *State whether a whole brain scan was used OR define the area of acquisition, describing how the region was determined.*

Diffusion MRI ☐ Used ☐ Not used

## Preprocessing

Preprocessing software | *Provide detail on software version and revision number and on specific parameters (model/functions, brain extraction, segmentation, smoothing kernel size, etc.).*

Normalization | *If data were normalized/standardized, describe the approach(es): specify linear or non-linear and define image types used for transformation OR indicate that data were not normalized and explain rationale for lack of normalization.*

Normalization template | *Describe the template used for normalization/transformation, specifying subject space or group standardized space (e.g. original Talairach, MNI305, ICBM152) OR indicate that the data were not normalized.*

Noise and artifact removal | *Describe your procedure(s) for artifact and structured noise removal, specifying motion parameters, tissue signals and physiological signals (heart rate, respiration).*

Volume censoring | *Define your software and/or method and criteria for volume censoring, and state the extent of such censoring.*

## Statistical modeling & inference

Model type and settings | *Specify type (mass univariate, multivariate, RSA, predictive, etc.) and describe essential details of the model at the first and second levels (e.g. fixed, random or mixed effects; drift or auto-correlation).*

Effect(s) tested | *Define precise effect in terms of the task or stimulus conditions instead of psychological concepts and indicate whether ANOVA or factorial designs were used.*

Specify type of analysis: ☐ Whole brain ☐ ROI-based ☐ Both

Statistic type for inference | *Specify voxel-wise or cluster-wise and report all relevant parameters for cluster-wise methods.*

(See Eklund et al. 2016)

Correction | *Describe the type of correction and how it is obtained for multiple comparisons (e.g. FWE, FDR, permutation or Monte Carlo).*

## Models & analysis

n/a | Involved in the study

- [ ] [ ] Functional and/or effective connectivity
- [ ] [ ] Graph analysis
- [ ] [ ] Multivariate modeling or predictive analysis

| | |
|---|---|
| Functional and/or effective connectivity | *Report the measures of dependence used and the model details (e.g. Pearson correlation, partial correlation, mutual information).* |
| Graph analysis | *Report the dependent variable and connectivity measure, specifying weighted graph or binarized graph, subject- or group-level, and the global and/or node summaries used (e.g. clustering coefficient, efficiency, etc.).* |
| Multivariate modeling and predictive analysis | *Specify independent variables, features extraction and dimension reduction, model, training and evaluation metrics.* |
